# Stevens-Johnson Syndrome and Toxic Epidermal Necrolysis Overlap Induced by Trimethoprim/Sulfamethoxazole: A Case Report

**DOI:** 10.7759/cureus.108225

**Published:** 2026-05-04

**Authors:** Guilherme Kenzo Shimizu Saito, Gabriela Mendes da Costa Manteli, Beatris Pinheiro dos Santos, Lucas Garrafoni Pessotti, Stefany Laura Messias, Lara Cordeiro de Faria, Juliana Adachi, Andréa Paula Peneluppi de Medeiros

**Affiliations:** 1 Department of Internal Medicine, Hospital Municipal Universitário de Taubaté, Taubaté, BRA; 2 Department of Preventive Medicine, Hospital Municipal Universitário de Taubaté, Taubaté, BRA

**Keywords:** clinical dermatology, drug-related side effects and adverse reactions, stevens-johnson syndrome (sjs), toxic epidermal necrolysis (ten), trimethoprim-sulfamethoxazole

## Abstract

Stevens-Johnson syndrome (SJS) and toxic epidermal necrolysis (TEN) overlap is a rare, potentially life-threatening cutaneous hypersensitivity reaction that requires prompt recognition and appropriate treatment. Drug-induced hypersensitivity reactions are commonly associated with medications such as antimicrobials, anticonvulsants, and nonsteroidal anti-inflammatory drugs. This case report describes an 18-year-old female patient who was prescribed trimethoprim/sulfamethoxazole for asymptomatic bacteriuria in a primary care setting, despite the absence of a clear clinical indication, and subsequently developed SJS/TEN overlap with epidermal detachment involving 20% of the body surface area. The patient improved after withdrawal of the offending agent and intensive supportive management and was discharged after 22 days of hospitalization. This case highlights the importance of early diagnosis and immediate drug discontinuation to improve outcomes.

## Introduction

Stevens-Johnson syndrome (SJS), toxic epidermal necrolysis (TEN), and SJS/TEN overlap are considered variants within the same spectrum of epidermal necrolysis, primarily distinguished by the extent of body surface area (BSA) affected by epidermal detachment [1-4]. These conditions are rare, with an estimated annual incidence of 1-10 cases per million population [2,3,5]. They constitute dermatologic emergencies characterized by acute epidermal loss resembling extensive burns, predisposing patients to dehydration, infection, systemic complications, and death. SJS is characterized by detachment involving less than 10% of the BSA, whereas TEN involves detachment of more than 30%. Detachment affecting 10-30% of the BSA is classified as SJS/TEN overlap [1,2].

Although diagnosis is primarily clinical, a skin biopsy is the preferred method for confirmation, particularly in atypical cases or when excluding differential diagnoses. Histopathological findings may include full-thickness epidermal necrosis, necrotic keratinocytes, sparse mononuclear dermal infiltrate, and negative direct immunofluorescence, supporting the diagnosis of severe drug-induced cutaneous adverse reactions [1,3]. Clinically, a prodromal flu-like phase with fever and malaise may precede the appearance of irregular erythematous macules, atypical target lesions, flaccid blisters, and mucosal involvement (ocular, oral, and/or genital) in up to 80% of cases [1,2]. Moreover, mortality rates in SJS/TEN vary substantially according to prognostic severity markers, such as the Score of Toxic Epidermal Necrolysis (SCORTEN), and may exceed 30% in high-risk patients [1,2].

SJS/TEN overlap is most commonly a drug-induced, immune-mediated disorder. Medications frequently implicated include antimicrobials, anticonvulsants, and nonsteroidal anti-inflammatory drugs. Among antimicrobials, trimethoprim/sulfamethoxazole (TMP-SMX) is a well-recognized high-risk trigger of drug-induced dermatoses and remains one of the agents most frequently implicated in these reactions [2,5,6]. Accordingly, management fundamentally involves the immediate discontinuation of the suspected drug and intensive supportive care, including fluid and electrolyte replacement, wound and skin care, pain control, nutritional support, infection prevention, and multidisciplinary involvement [2,6].

## Case presentation

An 18-year-old female patient, nonpregnant and previously healthy, born and residing in the interior of the state of São Paulo, Brazil, was started on TMP-SMX (160/800 mg every 12 hours for 14 days) for asymptomatic bacteriuria detected on a routine urinalysis in primary care, despite the absence of a clear clinical indication. After three days of medication use, she developed cutaneous erythema, malaise, and headache. In this context, she sought care at an urgent care unit, where she was prescribed an oral corticosteroid for home use. At that time, no recommendation was made to discontinue TMP-SMX.

One week after treatment initiation, while still using TMP-SMX, the patient developed clinical deterioration, generalized erythema, epidermal detachment involving multiple body sites, and painful mucosal lesions. She again sought care at an urgent care unit and was then admitted to the emergency department, where the recent drug exposure associated with the acute onset of a widespread erythematous rash, skin detachment, and mucosal involvement raised diagnostic suspicion of SJS/TEN overlap. The patient was subsequently transferred to the intensive care unit (ICU) of a tertiary hospital.

Upon ICU admission, the patient presented with severe dehydration and the following vital signs: blood pressure at 102/76 mmHg; heart rate at 121 beats per minute (bpm); respiratory rate at 19 breaths/min; and oxygen saturation at 96% on room air. Initial laboratory evaluation demonstrated low hemoglobin and hematocrit levels, with preserved leukocyte and platelet counts, elevated C-reactive protein, and serum electrolytes, renal function, and hepatic profile within normal limits (Table 1).

**Table 1:** Admission laboratory investigations. The laboratory tests were obtained one week after symptom onset, upon ICU admission.

Laboratory Test	Result	Normal Reference Range
Hemoglobin	9.5 g/dL	12.0-16.0 g/dL
Hematocrit	28%	36-48%
White blood cell count	5,100/µL	4,000-10,000/µL
Total platelets count	297,000/µL	150,000-450,000/µL
C-reactive protein	23.8 mg/L	<5 mg/L
Glucose	98 mg/dL	70-99 mg/dL
Blood urea	21 mg/dL	15-40 mg/dL
Creatinine	0.67 mg/dL	0.5-1.1 mg/dL
Sodium	141 mEq/L	135-145 mEq/L
Potassium	4.3 mEq/L	3.5-5.0 mEq/L
Alanine aminotransferase	23 U/L	7-35 U/L
Aspartate aminotransferase	32 U/L	10-35 U/L
Total bilirubin	0.2 mg/dL	0.2-1.2 mg/dL
Direct bilirubin	0.1 mg/dL	0.0-0.3 mg/dL

**Figure 1:**
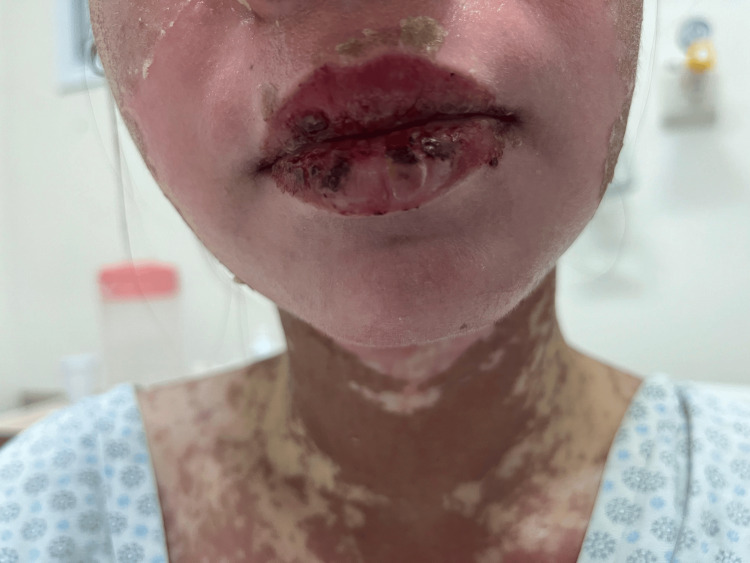
Lips showing erythema, edema, and hemorrhagic crusts, consistent with mucosal involvement in Stevens-Johnson syndrome/toxic epidermal necrolysis overlap. The photograph was obtained one week after symptom onset, upon ICU admission.

**Figure 2:**
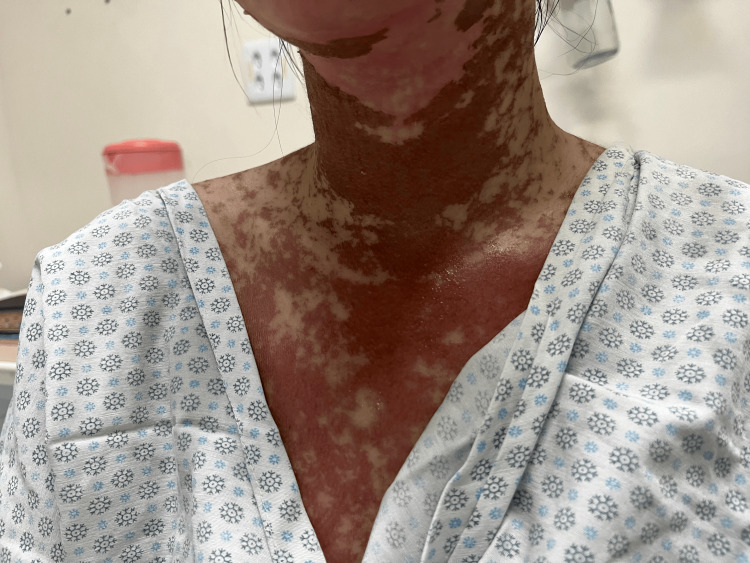
Cervical and anterior chest showing multiple erythematous-violaceous plaques with desquamative and dyschromic areas interspersed with spared skin, consistent with Stevens-Johnson syndrome/toxic epidermal necrolysis overlap. The photograph was obtained one week after symptom onset, upon ICU admission.

**Figure 3:**
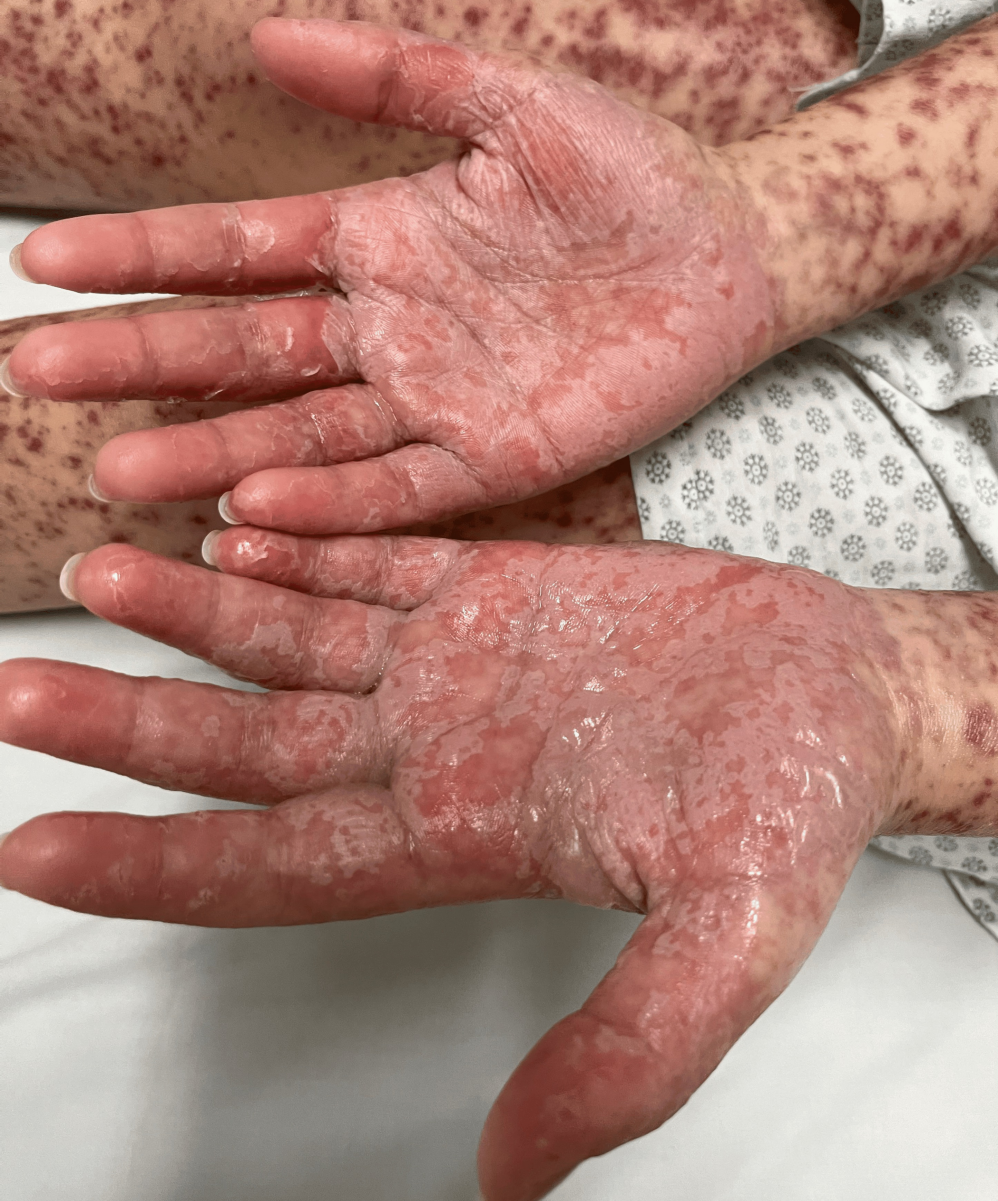
Bilateral palmar surfaces showing desquamative areas and skin detachment, characteristic of drug-induced dermatoses. The photograph was obtained one week after symptom onset, upon ICU admission.

**Figure 4:**
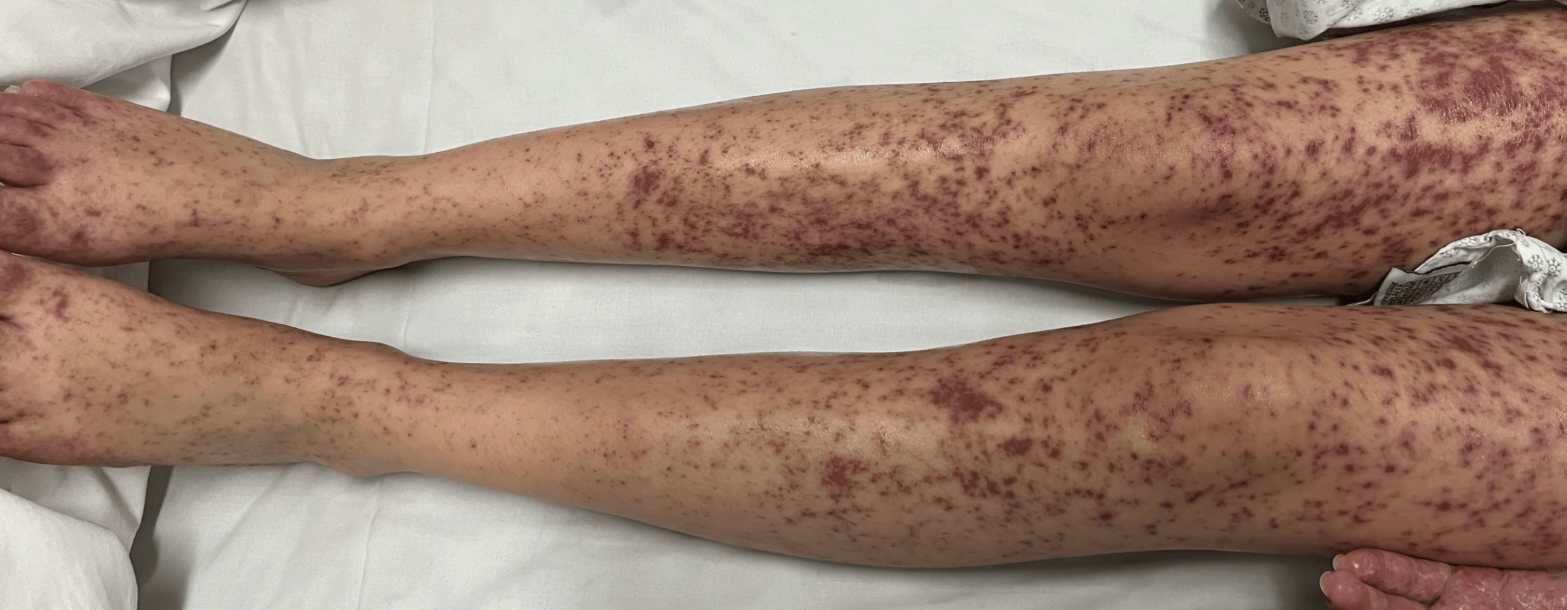
Lower extremities showing multiple erythematous-violaceous plaques with desquamative and dyschromic areas interspersed with spared skin, consistent with Stevens-Johnson syndrome/toxic epidermal necrolysis overlap. The photograph was obtained one week after symptom onset, upon ICU admission.

**Figure 5:**
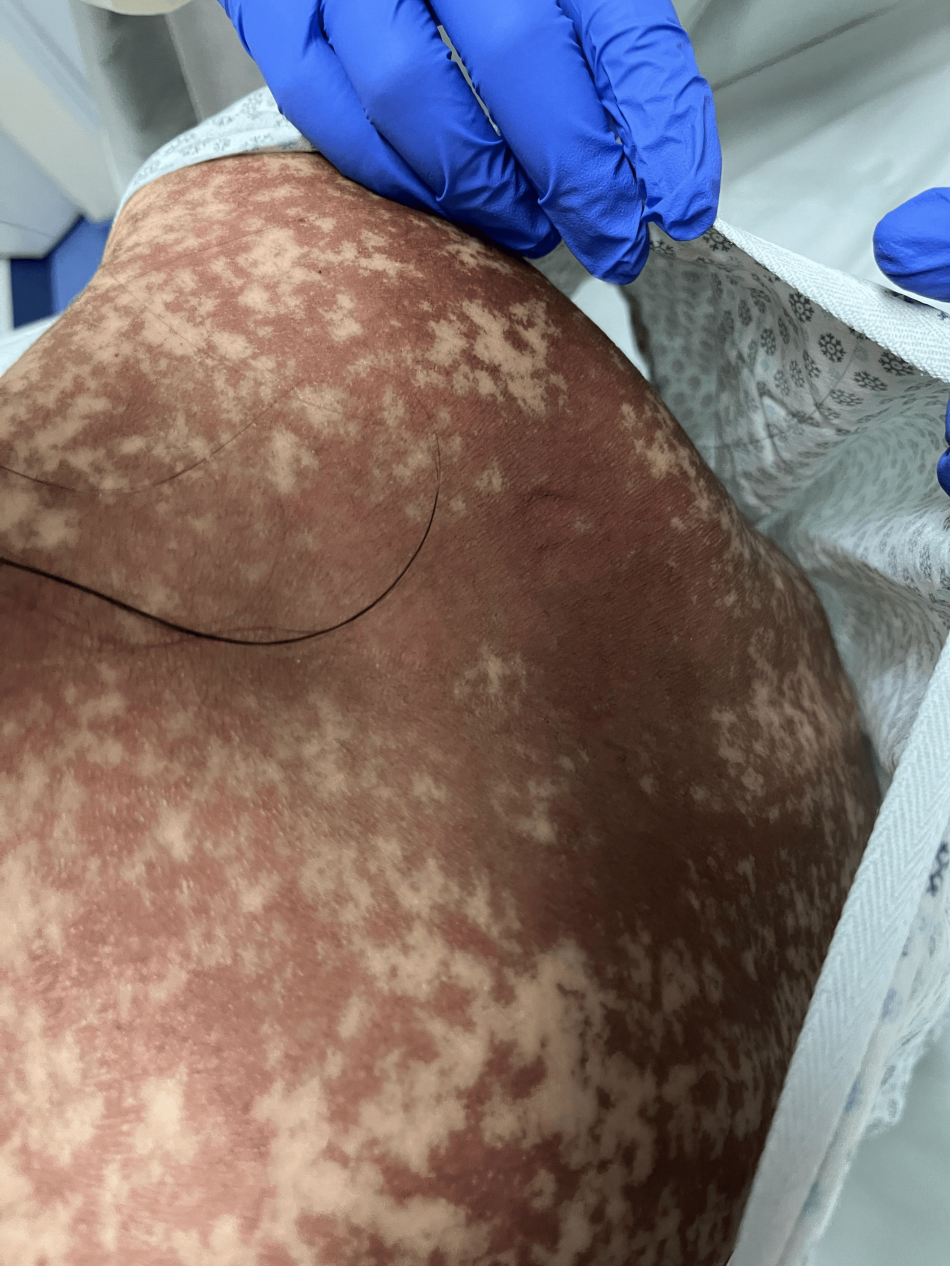
Dorsal region showing multiple erythematous-violaceous plaques with desquamative and dyschromic areas interspersed with spared skin, consistent with Stevens-Johnson syndrome/toxic epidermal necrolysis overlap. The photograph was obtained one week after symptom onset, upon ICU admission.

Dermatologic examination revealed widespread atypical erythematous-violaceous macules and plaques, partially confluent, associated with flaccid bullae, erosions, and epidermal detachment involving approximately 20% of the BSA, consistent with SJS/TEN overlap (Figures 1-5). Skin biopsy was not performed, as the diagnosis was considered clinically evident based on the characteristic presentation and temporal association with recent drug exposure.

At this time, TMP-SMX was discontinued, and advanced supportive care was initiated. Retrospective drug causality assessment using the algorithm of drug causality for epidermal necrolysis (ALDEN) score supported a probable causal relationship (ALDEN score = 4) between TMP-SMX exposure and the development of SJS/TEN overlap. The dermatology service was consulted, and resumption of corticosteroid therapy was recommended with intravenous methylprednisolone at a dose of 500 mg/day for five days, along with the liberal use of topical petroleum jelly or sunflower oil for hydration, protection, and recovery of the affected skin. Empirical antibiotic therapy with ceftriaxone was also initiated due to a suspected secondary cutaneous infection.

On the second day of ICU hospitalization, the ophthalmology and gynecology services were also consulted to evaluate possible ocular and genital mucosal involvement, respectively, with no involvement initially identified. Retrospective prognostic assessment using the SCORTEN yielded two points (heart rate >120 bpm and epidermal detachment >10% BSA), corresponding to an intermediate predicted mortality risk. The patient progressed favorably with the instituted supportive treatment, with stabilization of the condition and improvement in general status, and was discharged after 22 days of hospitalization.

At the 30-day post-discharge outpatient follow-up with the dermatology service, the patient remained clinically stable, with complete re-epithelialization and no residual sequelae.

## Discussion

SJS/TEN overlap should be considered in patients presenting with acute-onset mucocutaneous manifestations following the introduction of a new medication. Given its delayed immunologically mediated pathophysiology, symptoms typically arise within four to 28 days after exposure to the offending agent, most commonly within one to three weeks [1,2], a time course that is consistent with the present case and further supports the diagnosis. The disease results from a type IV hypersensitivity reaction in which the drug-peptide complex activates CD4+ T lymphocytes, cytotoxic CD8+ T cells, and natural killer cells, inducing keratinocyte apoptosis. This process culminates in full-thickness epidermal necrosis, leading to separation of the epidermis from the dermis [1,2,4].

Disease severity depends on the individual's sensitivity to the triggering medication, exhibiting a progression of dermatologic manifestations, including scenarios with multiple macules that may be either isolated or coalescent, evolving into vasculitis/purpura with epidermal detachment and blister formation [1-3,7]. The diagnosis of SJS/TEN overlap is based on the patient's clinical presentation, considering the morphological pattern of the lesions, the distribution of dermatologic manifestations, and the percentage of epidermal detachment. In this context, involvement of 10-30% of the BSA is observed [1,2,8], as in the present case.

Moreover, mucosal involvement during the course of SJS/TEN overlap often results in additional complications, such as the development of synechiae, particularly in the ocular conjunctiva and oral mucosa, compromising their function [8]. In addition, affected individuals may develop cutaneous sequelae, including atrophic or hypertrophic scars, keloids, and post-inflammatory dyschromia. Cicatricial alopecia and onychodystrophy have also been reported [8-10]. These complications are not limited to physical or functional aspects but are also closely associated with psychological distress resulting from the aesthetic and social impact of the disease [9,10].

Considering the causative role of TMP-SMX in the present case, it is important to emphasize that this agent is a well-recognized trigger of SJS/TEN overlap. Although generally effective and widely prescribed, its use should be restricted to evidence-based indications, as unnecessary exposure may increase the risk of severe cutaneous adverse reactions [3,11]. Genetic susceptibility also appears to contribute to disease development. Specific human leukocyte antigen (HLA) alleles have been associated with increased risk of severe cutaneous adverse reactions to several medications, and emerging evidence suggests that certain HLA variants may also predispose individuals to TMP-SMX-induced dermatoses [12]. Several previously published reports have likewise identified TMP-SMX as an important antimicrobial trigger of SJS/TEN overlap, often affecting young and otherwise healthy individuals, with symptom onset typically occurring within the first one to three weeks after treatment initiation [3,11,12]. Similar to those reports, the present patient developed prodromal symptoms, followed by rapidly progressive mucocutaneous involvement after recent TMP-SMX exposure.

It is noteworthy that the morbidity and mortality of SJS/TEN overlap are comparable to those observed in patients with extensive burns, reflecting a guarded prognosis [10]. In this context, the SCORTEN is a validated prognostic tool widely used to estimate mortality in affected patients, based on clinical and laboratory parameters assessed within the first 24 hours of admission. Its application allows early risk stratification and may inform clinical decision-making [5]. In the present case, a retrospective SCORTEN assessment yielded two points, corresponding to an intermediate predicted mortality risk. In addition, earlier discontinuation of TMP-SMX at the time of the first clinical presentation, when erythema and systemic symptoms had already emerged, might have limited disease progression and reduced the extent of epidermal detachment. Early withdrawal of the offending agent remains one of the most important modifiable prognostic factors in SJS/TEN overlap [1-4,6]. Therefore, early diagnosis of severe drug-induced cutaneous reactions, with immediate withdrawal of the suspected medication, followed by close monitoring and intensive supportive care, is essential in the disease course and, consequently, in patient outcomes [1,2,10].

## Conclusions

SJS/TEN overlap is a severe and potentially life-threatening condition that requires early recognition. General practitioners and dermatologists should maintain a high index of suspicion, particularly after recent exposure to new medications. In the present case, failure to discontinue TMP-SMX at the initial presentation of erythema and systemic symptoms was followed by clinical progression, highlighting how delayed recognition and continued exposure to the offending drug may worsen disease severity. Prompt medication review and immediate drug discontinuation are therefore essential. The SCORTEN should also be considered early in the evaluation process, as it provides valuable prognostic information and may support risk stratification in both clinical practice and future research. Careful monitoring of complications, intensive supportive care, and multidisciplinary follow-up are fundamental to optimizing outcomes and reducing morbidity and mortality.
